# Predicting In Vitro Neurotoxicity Induced by Nanoparticles Using Machine Learning

**DOI:** 10.3390/ijms21155280

**Published:** 2020-07-25

**Authors:** Irini Furxhi, Finbarr Murphy

**Affiliations:** Transgero Limited, V42V384 Newcastle, Limerick, Ireland; finbarr.murphy@transgero.eu

**Keywords:** neurotoxicity, in vitro, machine learning, nanotoxicology

## Abstract

The practice of non-testing approaches in nanoparticles hazard assessment is necessary to identify and classify potential risks in a cost effective and timely manner. Machine learning techniques have been applied in the field of nanotoxicology with encouraging results. A neurotoxicity classification model for diverse nanoparticles is presented in this study. A data set created from multiple literature sources consisting of nanoparticles physicochemical properties, exposure conditions and in vitro characteristics is compiled to predict cell viability. Pre-processing techniques were applied such as normalization methods and two supervised instance methods, a synthetic minority over-sampling technique to address biased predictions and production of subsamples via bootstrapping. The classification model was developed using random forest and goodness-of-fit with additional robustness and predictability metrics were used to evaluate the performance. Information gain analysis identified the exposure dose and duration, toxicological assay, cell type, and zeta potential as the five most important attributes to predict neurotoxicity in vitro. This is the first tissue-specific machine learning tool for neurotoxicity prediction caused by nanoparticles in in vitro systems. The model performs better than non-tissue specific models.

## 1. Introduction

The rise of nanotechnology and rapid production of nanoscale materials have increased human and ecosystem exposure to NanoParticles (NPs). Determining the potential hazards of NPs is therefore essential for both protecting organism health and ensuring the benefits of nanoenabled products. Nanotoxicology (the study of NPs toxicity) has been a topic of rigorous research for more than 20 years [1]. Many factors that affect NPs toxicity and the underlying mechanisms have been investigated [2]. Surface chemical components, for instance, can cause Reactive Oxygen Species (ROS), which can induce oxidative stress, resulting in disturbed physiological redox-regulated functions inside a cell. This in turn may lead to DNA damage, unregulated cell signaling, cytotoxicity, apoptosis, and cancer initiation [3]. Surface coating, size, morphology, surface charge, and other physicochemical (p-chem) properties have all been shown to affect NPs toxicity [1,4,5,6].

Traditional hazard assessment relies mostly on in vivo testing. The Organisation for Economic Co-operation and Development (OECD) for instance recommends a series of test guidelines for acute/subchronic/chronic assessment [7] and developmental neurotoxicity [8]. Data generated with these protocols are relevant and reliable for the assessment of those specific endpoints [9], but are of high-cost and low time efficiency. In vitro and in silico tests are alternatives for fulfilling safety assessments considering the increasing number of NPs [10,11]. Whilst guidelines to facilitate the interpretation of toxicological results for harmonized in vitro methods have been issued [12], as of yet there have been no guidelines for in silico approaches [13]. However, a number of explanatory documents and reports have been produced by various regulatory agencies that provide leadership on how to use and report in silico approaches (for example what constitutes a valid model; what is it appropriate usage; what serves as sufficient documentation) [14].

In silico methods are gaining popularity in accordance with the 3R (Replacement, Reduction and Refinement) principles of diminishing in vivo studies and are increasingly cited within regulatory frameworks as ways to fulfil data requirements. Although the Registration, Evaluation, Authorisation and Restriction of Chemicals regulation (REACH) defends implementing such alternative approaches as exploratory or predictive tools in hazard assessment [15], those tools are accepted by regulators as complementary rather than stand-alone methods [16,17].

Machine Learning (ML) is a method of data analysis that automates analytical model building. It is a branch of artificial intelligence based on the idea that systems can learn from data and identify patterns. Diverse ML tools have been developed during the last two decades that explore the prediction of toxicological properties or the adverse effects of NPs [18]. Quantitative structure−activity/toxicity relationships (QSARs/ QSPRs) are among the most widely used practices [19]. ML does not require deterministic insights; bypassing in depth comprehension of the interactions within a system, it constructs a computational predictor bridging input data directly to the outcome. Furthermore, those tools are fast and cheap, and as they rely on information inputs rather than physical test materials, can be used to predict the impact of materials not yet synthesized, thereby contributing to safe-by-design approaches [20].

The majority of the ML tools do not predict endpoints that are relevant for regulatory purposes such as carcinogenicity, mutagenicity or acute toxicity [18]. Cell viability and aggregated endpoints amongst others, have been popular toxicological predictors with in silico tools in the field of nanotoxicology. Most of the tools are built on data from in vitro studies derived from diverse tissues (lung, skin, etc.). A small number of models use in vitro experimental information such as cell origin (human, rodent etc.), cell lines (lung, skin, pancreas etc.) or cell name as input variables [18]. Few models in the literature capture brain tissue in their modelling:

- Trinh, et al. [21] used in vitro characteristics as input parameters (cell line, origin, type etc.,) where Glioma, Brain Microvascular Endothelial Cells (BMEC) and neuroblastoma cell lines (SHSY5Y) derived from different species (human and murine) were included amongst other cell lines. Their focus was curation and meta-analysis of data regarding metallic (Au and Ag) NPs for the prediction of cytotoxicity using decision trees and instance-based algorithms.
- Labouta, et al. [22] developed decision trees for assembling information on cytotoxicity of several NPs considering NP-features, cell-related features (cell-type, cell line, organism, organ/tissue source, age and morphology) as well as methodological parameters related to cytotoxicity/cell viability test and the exposure time. Human epithelial and mouse neuronal cell lines were included as a representation of brain tissue amongst other organs.
- Ha, et al. [23] performed a meta-analysis of published articles on oxide NPs using attributes of p-chem, toxicological, and quantum-mechanical properties. Brain tissue was considered in their analysis for the prediction of cell viability in a binary form using trees algorithms.
- Bilal, et al. [24] and Oh, et al. [25] built ML tools based on trees and bayes algorithms for exploring the cellular toxicity of cadmium-containing quantum dots. Models were developed based on a dataset compiled from publications comprising cell viability and half maximal inhibitory concentration (IC50) from in vitro data samples. Among many variables, they included cell anatomical type (neural, neuronal) and cell name (i.e., PC12, N9) with different origins (human, rat, mouse), while specifying the tissue in detail (spine, brain, adrenal-gland and hypothalamus).
- Marvin, et al. [26] built a Bayesian Network for the prediction of an aggregated hazard outcome based on eight biological effects, namely, neurological, cardio-pulmonary, immunological, inflammation, genotoxicity, reaches central nervous system; fibrosis and cytotoxicity using p-chem characteristics and information regarding the study type (in vitro or in vivo).
- Furxhi, et al. [20] built Bayesian Networks bridging NP p-chem properties, experimental exposure conditions and in vitro characteristics with biological effects of NPs on a molecular cellular level from transcriptomics studies. Regarding brain tissue, the SHSY5Y cell line was used as a model input variable.

To our knowledge, there is neither an explicit mechanistic interpretation nor a non-mechanistic predictive model *specific* to a neurotoxicological outcome. Brain as a tissue variable or a neurotoxicological endpoint has been used in in silico tools in combination with other cell lines or in combination with other outcomes [18]. Aggregated in such a way, this information is easily neglected by data-seekers and is retrievable only when sought for in Supplementary Materials.

This study develops a ML classifier that predicts solely neurotoxicological, NP-induced, cellular viability using inputs addressing p-chem properties, experimental exposure conditions and in vitro characteristics. It gathers all aforementioned brain tissue toxicity experimental literature data in one complete dataset. It uses the data to build a predictive model that is biologically accurate, i.e., tissue-specific, but not biologically deduced. ML allows bypassing the uncertainty of input estimation in applying physiology-based mass transport models. Furthermore, the ML model explicitly considers in vitro experimental conditions, such as exposure dose, duration or toxicological assay, as input parameters and, therefore, can be applied for diverse in vitro brain tissue exposures. The model is validated accordingly and, where possible, compared to previous works. Our main motivation is that in silico tools, for the prediction of a target-tissue effect, should be biologically and toxicologically specific and aim at a definite endpoint. This approach has the advantage of focusing on a target tissue/organ response at the cellular level since, compared to multiple tissue combining approaches, it exhibits improved toxicity predictivity.

## 2. Materials and Methods

**Figure 1:**
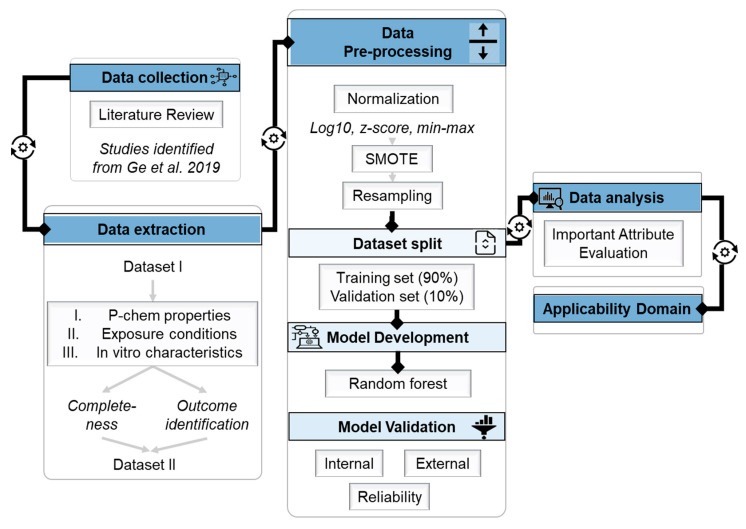
Model development workflow.

Figure 1 shows the workflow for the model implementation. Initially, studies assessing neurotoxicity of NPs in vitro were identified (Section 2.1). Information regarding p-chem properties, exposure conditions, in vitro characteristics (input variables), and endpoints (outputs) was extracted and data completeness was assessed. A data set (Dataset I) comprised of 895 observations was created at first with multiple outcomes. After identifying the final output to be predicted, based on data completeness, Dataset II was formed, processed and used to develop the model, comprised of 603 rows (Section 2.2). Various normalization methods were carried out, a Synthetic Minority Oversampling Technique (SMOTE) was applied to increase model performance and to minimize prediction biases, and bootstrapping was applied to further facilitate model application. Multiple random splits were performed and the training datasets were used for model development using a *trees* algorithm, random forest (RF). The model is evaluated internally and externally via metrics based on the confusion matrix. Furthermore, additional toxicity data identified from a meta-analysis of nanoparticle cytotoxicity were used for reliability validation of the developed model (Section 2.4). Analysis of important attributes based on model information gain (entropy) was conducted to reveal the most significant inputs regarding outcome prediction (Section 2.5). Finally, the applicability domain was defined (Section 2.6).

### 2.1. Data Collection

Studies of NP-induced neurotoxicity were assembled in a recent review paper by [27]. The paper (*ibid*) reviewed studies with an emphasis on molecular and cellular mechanisms. The authors concluded that NPs induce oxidative stress, inflammation, DNA damage, and cell death, all of which are potential mechanisms of tissue toxicity. From the reviewed studies, we focused on 36 articles referring only to in vitro experiments. Having in vivo and in vitro data in one dataset requires finding a harmonized way of reporting dose metrics in different systems, a complex and challenging topic not addressed here. For example, in vivo studies report the exposure dose in mg/kg [28] or mg/kg bw/day [29] with multiple durations and frequencies of exposure [30]. On the other hand, in in vitro assays, single dose is expressed as μg/mL in most of the cases. Harmonizing those dose metrics would result in assumptions feeding uncertainty to a model.

### 2.2. Data Extraction

Dataset I—Extraction of outcomes

From each study reviewed, several outcomes (mechanisms of NP-induced neurotoxicity in vitro) were recorded in accordance with [27]. Those outcomes are summarized in Table 1.

The above outcomes were extracted in a binary form (toxic, non-toxic). If a study determined that one particle was more toxic than another one, this is not shown in binary outcomes. Both particles are presented as toxic without demonstrating the relative toxicities among them. Only statistically significant alterations were extracted as toxic instances. For instance, if cell viability was examined in an assay using different exposure doses, only the statistically significant cases (less than 50% of viable cells) were recorded as toxic, while the rest of the instances were recorded as non-toxic. Studies demonstrated an outcome with specific exposure scenarios, for example exposure *A* and duration *B* resulted in outcome *C*. All information was extracted in instances (rows); if outcome (*C*) remained, the same during different exposure conditions (*A_1,2…n_, B_1,2…n_*), this resulted in multiple instance cases in our worksheet. However, a study might have applied various toxicological assays (diverse endpoints) for the same NP. In this case, if the study demonstrated that the NP is non-toxic for a specific outcome (i.e., DNA damage), information could not be assumed for the other outcomes e.g., membrane integrity, resulting in missing information for that experiment concerning the other outcomes.

The toxicity of NPs can be determined by numerous factors such as the dose and duration as well as the p-chem properties. Furthermore, the experimental in vitro parameters i.e., the cell type (neuroblastoma, pheochromocytoma etc.), cell origin (human, rat, mouse etc.) and the assay (MTS, XTT etc.) add further variables that affect the outcome. There are no definite guidelines in dataset formation and model implementation to create a predictive model with nanotoxicological data. Nevertheless, prior research has demonstrated the significance of NP type, p-chem properties, exposure conditions, and experimental parameters in predictive toxicity modelling [20,24,31,32,33,34,35,36].

In this study, each paper was reviewed focusing on information related to (i) NP type (FeO, SiO_2_, CuO etc.), (ii) nano-specific descriptors (core size, shape, zeta potential etc.) and (iii) study design experimental parameters (exposure conditions and in vitro characteristics). The data was extracted to construct the input variables. Ge, Du, Ran, Liu, Wang, Ma, Cheng and Sun [20] recorded the NP type and some p-chem properties. However, after a detailed review of the studies, we identified several properties not mentioned in their (*ibid)* article, such as agglomeration/aggregation, shape, surface charge, hydrodynamic size, details on zeta potential (negative or positive potential was provided), dose, duration etc., While the review study provided the ground for the data extraction, we scanned the papers to extract all available information in greater details. Due to the high occurrence of missing data on outcomes and the lack of detailed biological knowledge of the relationship (causality) among the different outcomes, we chose to model the prediction of specific cell lines targeted on *neural system cellular viability*, in a binary form (toxic, non-toxic). Thus, the instances (rows) corresponding to the other outcomes were discharged in the new dataset (Dataset II).

### 2.3. Data Management

Following data extraction and cleansing, all data was gathered in a single dataset along with all relevant bibliographical, descriptive, and technical metadata. These include all publications and author information and the methods and assays identified during data curation. In accordance with the European Commission’s Open Data Policy and the FAIR (Findable, Accessible, Interoperable, Reusable) data principles, the resultant dataset (data and metadata) was semantically annotated using established ontologies (e.g., eNanoMapper ontology, Chemical Entities of Biological Interest ontology, National Centre for Biotechnology Information ontology). The dataset was uploaded and made available under Creative Commons Attribution 4.0 International (CC BY-NC 4.0) licence, through the NanoCommons Knowledge Base (available online: https://ssl.biomax.de/nanocommons/cgi/login_bioxm_portal.cgi, accessed on 15 June 2020).

### 2.4. Data Pre-Processing and Validation

As measurement units and magnitude range differ and can affect the optimization of the model during training, data was normalized [31]. Data normalization was conducted on the numeric inputs to enhance model performance [19]. We used different normalization techniques such as log10, z-score and min-max for each input individually while assessing the skewness. Skewness is a measure of symmetry in a distribution of data, and a good value lies between −2 and 2. To investigate models performance with the different normalization methods, we created three different datasets (i) numeric inputs as log10, (ii) numeric inputs normalized along min-max and (iii) numeric inputs normalized as z-score. WEKA (Waikato Environment for Knowledge Analysis, version 3.8.4, available online: https://www.cs.waikato.ac.nz/ml/weka/, accessed on 18 May 2020), an open-source Machine Learning workbench, was used to train RF algorithm in default mode. Details on default values in model configuration can be found at: https://weka.sourceforge.io/doc.dev/weka/classifiers/trees/RandomForest.html, accessed on 18 May 2020. RF has been found to perform very well compared to other algorithms and demonstrate high performance in cases of missing values [37].

A class imbalance problem occurs when one of the classes has more samples than the other. The performance of most classification algorithms can be limited by unbalanced data. To avoid compromising the performance, we balanced the dataset by adjusting the relative frequency of toxic/non-toxic instances through resampling the dataset applying SMOTE, a supervised instance algorithm that oversamples the minority instances using k-nearest-neighbour [20]. Once a balanced dataset was achieved, we bootstrapped the data to further facilitate model application. Bootstrapping in general refers to sampling of data with replacement maintaining the same data distribution [38,39]. In our cases, we implemented bootstrapping in two steps; first an oversampling was applied to the balanced dataset to increase dataset size; then, we randomly sampled the dataset 10 times to different sets of 90% (training dataset for internal validation) and 10% (external validation) of the data [19]. Oversampling was applied through a supervised filter that doubled the number of instances. Ten-fold sampling with replacement was applied though a supervised filter to ensure stratification i.e., balanced classes in the subsets [40]. It should be noted that, in contrast to typical *k*-fold cross-validation (CV), the 10 subsets were overlapping. For each of the 10 subsets, internal validation (goodness-of-fit, robustness) was done for the training subset using 10-fold CV. In *k*-fold CV, the training set is randomly split into *k* mutually exclusive subsets. The model is trained and tested k times, each time being used to make predictions for instances that were withheld from the training set. By bootstrapping the data followed by typical CV, we applied a nested cross validation able to reveal overfitting and to estimate how well a model accounts for the variance of the response in the training set (goodness-of-fit). In addition, using the cross validation, we ensure model stability of prediction when a perturbation is applied in the training set (robustness) [14]. For each of the 10 subsets, the remaining 10% of the data were used for external validation (predictivity).

Different performance metrics were applied based on the confusion matrix. We used diverse metrics since no single metric is optimal for model’s comparison [14]. Accuracy (ACC), sensitivity (SENS), specificity (SPEC), precision (PREC), F1-measure (F1), Receiver Operator Characteristic Curve (ROC), and Matthews Correlation Coefficient (MCC) were demonstrated. For a detailed explanation of the metrics please refer to the following articles, which also demonstrate the advantages of MCC metric when dealing with classification models [20,41,42,43].

### 2.5. Important Attribute Evaluation

Attribute importance can be relatively measured and quantified based on information obtained from the models. The advantage of measuring the importance based on built model information is that being closely tied to the model performance it incorporates the correlation structure between the predictors into the importance calculation [44]. We recorded the attribute importance of RF based on average impurity decrease (and number of nodes using that attribute) in WEKA via the information gain with respect to the outcome [45,46]. Information Gain measures the correlation between the attribute values and the class values estimating the entropy-based worth of each attribute. Values vary from 0 for no information, to 1 for maximum information.

### 2.6. Applicability Domain

The Applicability domain (AD) of a model defines mathematically the input space that corresponds to reliable model performance. AD calculation is based on comparing a test sample to the dimensional limitations of the training dataset. They are several approaches proposed to define the AD for toxicity models. The Bounding Box method defines the AD as the multidimensional rectangular space outlined by the minimum and maximum values of the input variables. It can also be used on the Principal Components of the data. Leverage is a distance method based on the Mahalanobis distance of the query nanoparticle from the centroid of the reference nanoparticle data. The k nearest neighbors’ approach (kNN) investigates the similarity of the test sample to the instances of the training dataset measuring the distances of the query nanoparticle to the nearest cases over a predefined threshold. Alternatively, the distance from the training set data centroid can be calculated. In order to eliminate any method-related bias, Bouncing box, bouncing box PCA, Leverage, Distance from centroid, kNN -fixed k and variable k, were used to examine whether the external validation dataset falls within the descriptor space [47].

When a dataset is comprised by non-numeric attributes, the applicability domain regarding those attributes is defined by the values present in the dataset. For instance, an experiment referring to a NP not already present in one of the studies comprising the training dataset falls out of the AD of the model.

## 3. Results

### 3.1. Data Extraction

**Figure 2:**
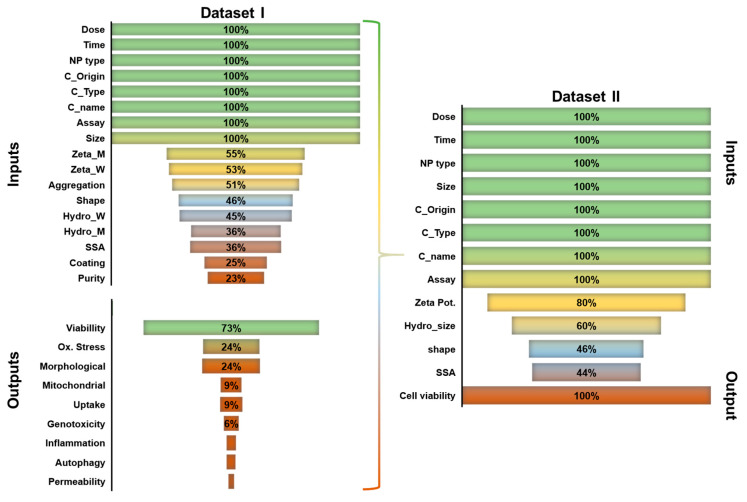
Dataset I completeness (percentages) of input parameters and the identified outcomes (**left**). Dataset II completeness of inputs with one outcome: cellular viability (**right**).

***Dataset I:*** From the studies reviewed several endpoints of NP-induced in vitro neurotoxicity were recorded in agreement to [20]. The initial dataset (Dataset I) was comprised of 895 rows derived from 39 studies focusing on the nervous system. The outcome of the fewest missing values was cellular viability with 73% completeness, as demonstrated in Figure 2 (left). Oxidative stress and cellular morphological changes were 24% of cases complete. Other endpoints, such as mitochondrial alterations, genotoxicity, pro-inflammatory responses etc., had insufficient completeness (<10%). This is attributed to the fact that studies assessing cellular viability used a high number of combinations of different exposure doses and/or cellular cultures, providing therefore most of the case. Figure 2 demonstrates the completeness of p-chem properties in Dataset I (left). Exposure dose and duration, NP type, cell origin, cell type, cell name, assay, and particle primary size were always reported in the studies, as expected. Zeta potential measured in different media (water, W, cellular medium, M) and aggregation were missing in ≈50% of the recorded instances. Completeness of shape, hydrodynamic size (W, M) and specific surface area was <50%. On the other hand, information regarding coating presence and purity percentages were scarcely mentioned in the studies. Due to low completeness of several outcomes, a model for the prediction of multiple outcomes would be poorly defined. In addition, due to the lack of detailed biological knowledge of the relationship (causality) among the different endpoints, we chose to create a specific in silico tool for the prediction of neurotoxic cellular viability in a binary form (toxic, non-toxic). Thus, the rest of the outcomes were discharged in the Dataset II. Since some rows did not provide information of cellular viability, but only for other endpoints, this resulted in a smaller dataset.

***Dataset II:*** Dataset II is comprised of 603 rows with p-chem properties, exposure conditions and in vitro characteristics to predict cellular viability derived from 32 studies. Compared to the original dataset, completeness was improved first, by deleting non-neurotoxic outcomes, and second, by grouping water and cell culture media measured attributes. Linear regression was used to estimate Zeta potential and Hydrodynamic size measured in water from values measured in cell medium, using data from the instances of the dataset where both values were measured. The improvement is presented in Figure 2 (right). Zeta potential, Hydrodynamic size and Surface Area completeness increased in comparison to Dataset I. No alterations were observed regarding shape. Coating, Aggregation, and Purity were discarded (<20% completeness, data not shown) and Zeta (M) and Hydrodynamic Size (M) were replaced in Dataset II.

The final 12 input variables: two exposure conditions, six p-chem properties, and four in vitro characteristics selected in our analysis are shown in Table 2. Cell viability (%) was classified as either toxic if cell viability was less than 50% and non-toxic otherwise.

### 3.2. Data Pre-Processing and Validation

**Figure 3:**
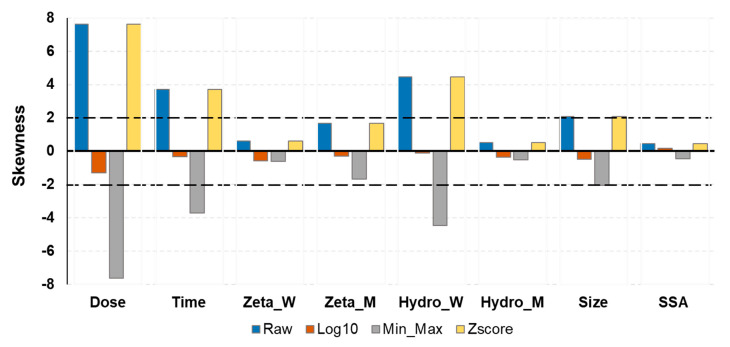
Skewness of numeric inputs based on different normalization methods. Four datasets were tested for their distribution of data, raw dataset (no normalization), log10, min-max, and zscore dataset.

Figure 3 demonstrates the data skewness of numerical inputs based on the three different normalization techniques (log10, min-max and z-score).

Inputs normalized with log10 had skewness closer to zero for all attributes, which means that the tails on both sides of the mean balance out overall. Raw dataset and mix-max had very high skewness. Z-score corrected some attributes but not all. The log10 dataset was chosen to be used in the RF model.

The balanced, normalized dataset was sampled randomly to 10 pairs of training and test sets with a ratio of 90:10; 90% was used for internal validation (goodness-of-fit, robustness) and 10% for external validation (predictability). The basic measures of the confusion matrix for internal and external validation for the 10 subsets are listed in Table 3.

An external dataset for reliability validation was extracted from a meta-analysis of NP cytotoxicity data [22]. The specific studies were selected based on specified cell-related attributes (brain tissue). From the extracted subsample from available Supplementary Materials, the reliability dataset is comprised of 210 rows. The instances identified are unique and not included in our analysis as the specific studies were not included in forming Datasets I and II. Exposure dose, duration, size, NP type, zeta potential, cell origin, cell name, cell type, assay, and viability were included in the dataset. Hydrodynamic size, specific surface area and shape were missing. Screening the source (study) of the instances, we found additional information, however, the missing values remained high for some instances e.g., 42%, 95% and 97% of values of zeta potential, hydrodynamic size and shape, respectively, missing.

The results of the reliability validation are shown in Table 3. The ACC results demonstrate the strength of the model to correctly capture the majority of the instances even if data of three input parameters are almost completely missing. Having a close look to the other metrics (MCC = 0.38), we see that the model was slightly biased towards the prediction of one class (nontoxic). The same is evident from the average SPEC results (66%) compared to 71% SENS.

### 3.3. Attribute Importance Evaluation

**Figure 4:**
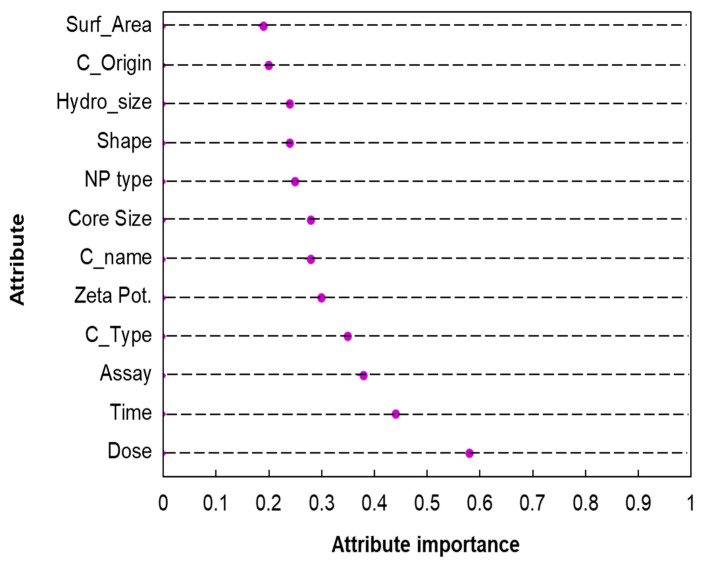
Attribute importance evaluation based on models’ information gain.

The results of attribute importance analysis are presented in Figure 4. Exposure dose and duration, toxicological assay, cell type, and zeta potential were identified as relatively important attributes when compared to the other attributes; this means that their values enable during classification the distinguishing of the materials as either toxic or non-toxic. In contrast, values of specific surface area, cell origin, hydrodynamic size, shape, and NP type had lower effect.

NP type, shape, core size, and cell name in general are expected to contribute to the change of the toxicity. However, the importance of the above attributes was measured relatively low in the analysis. The result does not mean that those attributes do not affect the toxicity change. Rather, it means that values of other attributes more decisively differentiate the outcome. It should be noted that the range or diversity of the attribute values affect the importance assessment. The model still uses the descriptive attributes such as NP type, shape and cell name to precisely predict the toxicity and builds on all available information from the attributes as a whole. Running the model without exposure conditions and in vitro characteristics resulted in lower Correctly Classified Instances (79%), and the information gain analysis showed the same pattern of physicochemical attributes affecting the prediction of outcomes i.e., zeta, core size and NP type as the most significant attributes. On the other hand, shape and surface area demonstrated lower information gain (data not shown).

### 3.4. Applicability Domain

Table 4 presents the portion of the external validation dataset that lies within the applicability domain of the model based on different methodologies regarding the numeric attribute data. All methods show that all instances of the dataset belong to the AD of the model, revealing a very balanced splitting. The result accords with the model performing equivalently for the internal and external validation.

Regarding nominal attributes, values of all external validation instances are included in the training dataset as well.

## 4. Discussion

In this study we presented a machine learning implementation, from data gathering to model validation, to predict neurotoxicity induced by NPs in in vitro systems. The model complies with the OECD principles [14] relating to: a defined endpoint; (cell viability is a biological effect that can be measured and therefore modelled); an unambiguous algorithm (details of models’ configuration are provided); a defined domain of applicability; appropriate measures of goodness-of-fit, robustness and predictivity (internal, external and reliability); and a mechanistic interpretation (assessment between input variables and endpoint via information gain).

**Figure 5:**
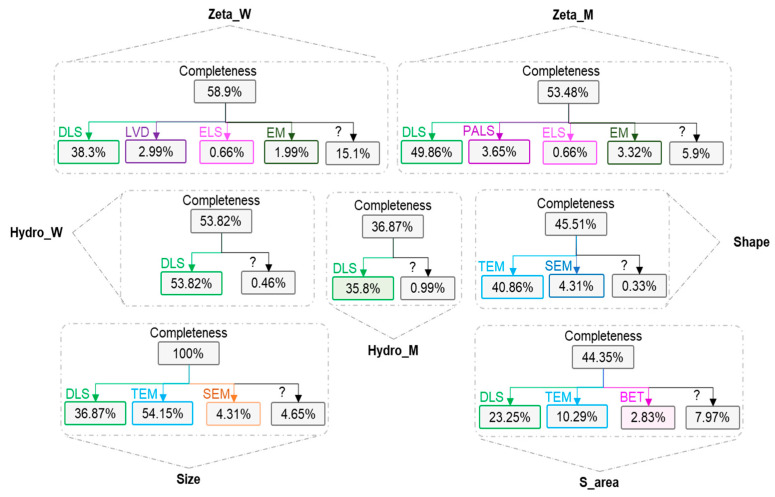
Physicochemical characterization completeness and experimental methodology.

Data should be developed from homogeneous datasets in which the experimental data have been generated by a single protocol. However, this is rarely viable in practice and data produced by different protocols are commonly combined. In order to capture experimental variations, the toxicological assay, serving as a surrogate of different protocols, is introduced to the model as an input variable. Regarding p-chem characterization measurements, most instances for Zeta (W, M), Hydro (W, M) and shape are measured with Dynamic Light Scattering (DLS). For zeta potential, other techniques were used, such as Laser Doppler Velocimetry (LDV), Electrophoretic Light Scattering (ELS), Electrophoretic mobility (EM) and Phase Analysis Light Scattering (PALS). Size was primarily measured with TEM (54%) which is also under potential ISO standardization and secondarily with DLS (37%), while few instances were determined through SEM. Surface area was determined mostly with DLS (23%), while some instances were identified with TEM and Brunauer–Emmett–Teller (BET). Data completeness is shown in Figure 5.

Mourdikoudis, et al. [48] provided an excellent review on characterization techniques mentioning strengths and limitations. The reproducibility of the p-chem characterization measurements is a tough quest, since NPs act as chameleons: they change with time, handling, and environmental conditions [49]. In this study, the experimental protocols were not taken under consideration. Future studies may integrate weighting scoring rules for each published article based on the measurement methods to evaluate the quality of the p-chem data as displayed in [21].

Few in silico tools in the literature capture neuronic cell cultures and do so only amongst other cell lines (see Table 5, Table 6 and Table 7). In contrast to those tools, we compiled, for the first time and in an organized manner, a toxicity dataset for the specific tissue. This resulted in a relatively small dataset but enabled focusing on impact induced by NPs only on the selected tissue as defined by the different variables related to the experimental study design and p-chem properties. The initial literature listing source was the review paper by [27], and the studies therein were examined for data extraction. Screening papers individually is highly time consuming. In this direction, efforts are being made to create curated harmonized databases, which will make the data FAIR. The FAIR Principles insist that all data be Findable, Accessible, Interoperable, and Reusable.

Among the studies capturing brain tissue, two studies retrieved data from the S2Nano (available online: http://portal.s2nano.org/, accessed on 23 July 2020) database (Table 5), while the rest practiced literature individual screening. Our dataset contains diverse NPs such as metal (Ag), metal oxides (ZnO, CuO, SiO_2_, etc.) and carbon-based NPs (SWCNT). From the tools available that capture neurotoxicity, two of them are trained for cadmium-containing semiconductor quantum dots, one is focused on metal NPs (Au, Ag), and the rest capture a variety of NPs, mostly focused on metals and metal oxides.

Our data are derived solely from in vitro studies, which is the practice in most tools (Table 5). One of the general limitations of in vitro test systems is that they are restricted to one or a few different cell types and, thus, cannot represent the biological responses in the whole organism. Single exposures are typically used in in vitro studies, which usually last from a few minutes up to a few days depending on the endpoint tested. Therefore, chronic exposures cannot be tested sufficiently. Defining a suitable dose range is also a challenge for both in vitro and in vivo tests, as often unrealistically high doses are chosen to observe an effect. Regarding the output, we predicted cell viability in binary form. Marvin, et al. [26] predicted an aggregated outcome (cytotoxicity, neurological, pulmonary, fibrosis, etc.) translated into a hazard band. Furxhi, Murphy, Poland, Sheehan, Mullins, and Mantecca [20] predicted disrupted pathways identified from transcriptomics studies in a cellular level. The rest of the studies predicted cell viability, which is the most common predicted endpoint in the field of nanotoxicology [18]. Diverse computational tools have been used, such as trees, instance base and bayes algorithms (Table 5).

In our study, numerous p-chem properties values were missing, which is a common issue in nanotoxicological studies. For example, surface area, hydrodynamic size and zeta potential measured in different media were absent in almost half our data samples. Nevertheless, RF has good performance even with missing values [37], highlighting the RF selection [19] and demonstrating the strength of ML tools to bypass missing knowledge. Researchers still encounter data shortness and lack of harmonized protocols, and theoretical understanding further complicates making data reproducible. Acknowledging the needs, the European Commission has embraced projects to explore the opportunities offered by modelling in coupling the toxicity and properties of NPs, such as GRACIOUS (available online: https://www.h2020gracious.eu/, accessed on 18 May 2020), eNanoMapper (available online: http://www.enanomapper.net/, accessed on 18 May 2020), NanoFASE (available online: http://www.nanofase.eu/, accessed on 18 May 2020), NanoCommons (available online: https://www.nanocommons.eu/, accessed on 18 May 2020), ACENano (available online: http://www.acenano-project.eu/, accessed on 18 May 2020), NanoInformaTIX (available online: http://www.nanoinformatix.eu/, accessed on 18 May 2020), and NanoSolveIT (available online: https://nanosolveit.eu/, accessed on 18 May 2020).

NP type (expressed as quantum dot core, NP type or NP material), particle primary size (expressed as diameter, particle size or core size) and zeta potential (surface charge) are used as input p-chem variables in all studies and this study as well (Table 6). Shape and specific surface area appear in three and four out of seven tools, respectively. In our study, zeta potential data measured in different media were initially gathered. However, due to low completeness, we integrated the variables into a single one. Only one study specified the medium in which zeta potential and hydrodynamic size were measured [21]. Besides p-chem properties, one study included quantum chemical properties in their dataset such as formation enthalpy, conduction band energy etc. [23] (Table 6).

Exposure conditions i.e., exposure dose and duration, are important attributes since the manifestation of adverse effect depends on exposure. Exposure conditions are included in six studies. Other exposure-related variables reported in some studies but not included in our study are delivery type and administration route (Table 6).

In vitro characteristics were included in our analysis i.e., cell origin/line/type and assay. Cell origin comprised of categories that indicate the species the cell culture is derived from (human, rat and mouse). Cell origin is used in three other studies termed as cell source species, cell species, and human/animal, while in one study, cell origin is used to describe whether cells are primary; cell type is used in half of the studies labelled as cell anatomical type or morphology; cell type is used in a study to describe if cell line is normal or cancerous. Cell line appears in four studies termed as cell identification or cell type. Finally, assay is used most of the time either as assay type, method or test. Those examples highlight the need for a harmonized way to describe the data. Brain tissue-related attributes information, which enabled performing a cross comparison of our tool with the existed ones, was retrieved from Supplementary Materials.

This suggests that looking for tools that cover e.g., a specific cell line cannot be limited to screening a study report, but requires going through the actual data samples and stresses the need for integrated databases.

The studies used in model implementation are considered biologically accurate, meaning that the model is trained with toxicological information manifested in cell lines derived only from the nervous system, as the potential target organ. In the best-case scenario, information would be derived only from human cell lines, but due to lack of data, cells lines from rodents were included. Moreover, the aggregation of different organ toxicities was not addressed, as this has been studied by a number of other investigators guided by the assumption that different tissue toxicities exhibit the same patterns that allow a totaled toxicity prediction.

Attribute importance analysis revealed that exposure dose, duration, assay, cell type, and zeta potential are the top five most important attributes affecting the prediction of neurotoxicity. The results are in accordance with the landscape presented in the field: Exposure dose and duration have been found as the most important attributes in all the studies covering brain tissue (Table 7). The assay of the toxicological measurement also appeared in the majority of studies as important, highlighting the significance of including experimental parameters in the dataset. In comparison with the other studies, the type of cell was found in the opposite spectrum of importance. This could be explained by the fact that our dataset being tissue-specific uncovered the fine influence on the outcome those types of cells possess. It should be noted that the aforementioned attributes are essentially the experimental parameters defining the study and not variables under investigation for their toxicity effect. Zeta potential appears in studies as important or otherwise; this signifies that the choice of all input variables influences the relative attribute importance. Only if the same set of variables is used across the studies, influential attributes analysis can be comparable and complementary.

Specific surface area, cell origin, hydrodynamic size, shape, and NP type are the five least important attributes affecting the prediction. Shape and cell origin appeared as such in other studies as well. Surface area appeared as either important or not, probably largely affected by low data completeness. NP type is found to be both highly and lowly influencing; Trinh, Ha, Choi, Byun and Yoon [21] had only one or two types of metal NP in their dataset, and this could explain why this variable provided little information in building the models. Particle size appeared as significant across all studies, highlighting the importance of size in the manifestation of toxicological effects. However, we did not find the same results when other non-p-chem attributes were included. When exposure conditions and in vitro characteristics were excluded, core size appeared the second most influential variable.

In conclusion, exposure conditions, assay and size are key parameters that enable the prediction of toxicity. Shape and cell origin do not influence toxicity prediction as much. Zeta potential, NP type and surface area have diverging behaviors. The comparison results indicate that key parameters are difficult to be determined in unison when each tool uses a different set of input variables.

We explored model performance in terms of robustness, goodness of fit and predictivity, in agreement with the fourth OECD principle. The performance was measured with metrics such as ACC, SENS, SPEC, MCC, ROC, and precision. MCC should be preferred in binary classification evaluation when compared to more basic metrics such as ACCU, SENS or SPEC [41]. In comparison with the other three models that predict cellular viability, our model shows better results, revealing that tissue-specific modelling enables better predictions. The studies did not demonstrate performance by a variety of metrics, making inter-comparison narrow. In details:

- Trinh, Ha, Choi, Byun and Yoon [21] trained five datasets different in terms of data qualities and degrees of completeness, for either a single NP or a combination of NPs. The model algorithms consist of RF and SVM. No internal validation metrics were provided, only external. The performance metrics included ACC, SENS, SPEC, and F1. The model with the highest ACC = 96.4% was SVM trained with AuNPs (dataset A1). This case reached SPEC = 100%, SENS = 50% and F1 = 0.50 showing that this model is biased towards the prediction of a specific class. When SENS reached 82.2% (SVM algorithm with A1 dataset), ACC dropped to 85% and F1 increased to 0.73. For the same metrics for external validation, our model reached ACC/SENS/SPEC = 98% and F1 = 0.98.
- Ha, Trinh, Choi, Maulina, Byun and Yoon [23] provided results of RF on five replications using four datasets (20 cases). The authors provided internal validation metrics derived from cross validation and external predictivity (ACC, SENS, F1 and precision). The most robust model reached ACC = 95%, SENS = 70%, F1 = 0.77 and PREC = 85% (Dataset III-A, 4th replication), lower than our case. The best predictivity was reached with another dataset (Dataset III-B, 3d replication) demonstrating ACC = 95%, SENS = 87%, F1 = 0.89 and 91% precision. In comparison, our model resulted in ACC/SENS/SPEC = 98% and F1 = 0.98.
- Labouta, Asgarian, Rinker and Cramb [22] trained eight decision trees internally using cross validation providing ACC, SENS and PREC. The best decision tree (DT4) achieved ACC = 91%, SENS = 98.3% and PREC = 92%. SENS is similar to the performance of our model, which appears more balanced with a 98% PREC.

Luan et al., Kleandrova et al. [35,36] and Concu et al. [50] built models based on perturbation theory using data from multiple literature sources to predict aggregated toxicological endpoints in a binary form for different NPs. Among the diverse in vitro characteristic inputs, they used brain tissues (Neuroblastoma, Neuro-2a cell line) amongst other mammalian lines with different origins. The perturbation models performed quite well. Those models were not included in the comparison, first, because the input variables consist of differentiations and combination of original attributes, making inter-comparison of attribute importance difficult, and second, the endpoint of prediction is an aggregated variable. However, even though the methodologies of data pre-processing are different, out model still compares well internally and externally.

Wrapping up, the tissue-specific, pre-processed RF model provides better predictions and performs adequately in reliability evaluation as well. Future studies should provide detailed and a plethora of metrics, internally and externally to facilitate comparison. No other study conducted a reliability validation.

Focusing on cellular viability specifically in brain tissue, the developed model performs better than models covering multiple tissues. When toxicities of different organs are merged in a ML classifier, tissue as an attribute appears to be a significant determinant of the output [20]. This comes as no surprise, since cells of various tissues differentiate both in structure, function, and therefore, toxicity. Even if the same outcome is selected, e.g., cell viability, and even if toxicities show similar patterns, the way the combinations of experimental conditions affect cell toxicity is not the same among different-tissue cells. Aggregating those cases in a ML approach without losing accuracy requires a versatile model and a significant number of data. If those cases are aggregated using the relatively small datasets available in nanotoxicity, different associations of NP characteristics, exposure conditions and toxicities are forced together, resulting in adulterated predictions.

Applicability domain, as a requirement of the OECD principles, was only identified in two studies [21,23]. The authors report the ranges of the numeric attributes calculated using k-nearest neighbours’ algorithm—weighted Euclidean distance. Similar to our study, the actual values of the nominal attributes are shown as defining the AD. In this way, a case of a NP present in the training dataset, injected in a specific biological assay also present in some other instance of the training dataset, is acceptable in the model AD. However, the reliability dataset cases not captured by our model showed that the exact combination of in vitro determinants of an experiment has to present in the training dataset. Assays, cell lines and cell types cannot be combined as independent variables in a classifier trained in small datasets, like the ones found in nanotoxicity. Although assays of the reliability cases, such as MTT or Alamar Blue and cell types, such as neuroblastoma or pheochromocytoma, can be found in the training set, their combinations are different among training and reliability sets, and the model fails to capture the outcomes. More data are needed to enable building models accounting accurately for the possible in vitro determinants’ combination. The same holds for NP p-chem characterization, as shape, zeta potential, and specific area data were scarce in the reliability dataset. Besides data availability, another point of dataset improvement is replacing NP type as an attribute with a representative cluster of theoretical descriptors. This would extend the AD of the model but burden the model complexity as training datasets remain small.

## 5. Conclusions

We developed a random forest model extracting data from multiple literature sources including experimental results, p-chem properties and exposure conditions. Various pre-processing techniques were used for model facilitation including analysis of relatively important attributes based on model information. The developed model predicts cellular viability specifically in brain tissue and performs better than models covering multiple tissues. Experimental conditions are shown to significantly affect predictions. In vitro determinants combinations must be treated with caution when training datasets are small.

## Figures and Tables

**Table 1:** Results of toxicological endpoints from in vitro neuronal assays, extracted as outcomes for use in developing the model (list of biological endpoints not exhaustive).

Mechanism and Results of Toxicity
Pro-inflammatory response (*e.g., cytokine production IL1β, IL-4, IL6, IL8, proteins or genes disruption linked with inflammatory processes*)
Mitochondrial effects (*e.g., depolarization, alterations in dynamics or morphology, loss of membrane potential*)
Cellular Uptake (*i.e., internalization*)
Autophagy (*e.g., reactions in autophagy process, alterations in phagocytosis activity, lysosomal activation*)
Cell viability (*i.e., cellular proliferation, mitochondrial activity, membrane integrity/damage, apoptosis induction*)
BBB permeability (*e.g., increased permeability, integrity*)
Genotoxicity (*e.g., DNA damage, disruption of synthesis*)
Oxidative stress (*i.e., ROS production, disrupted pathways or molecules, lipid peroxidation, antioxidant stimulation*)
Morphological changes (*i.e., damage-modification of cellular components or cellular form e.g., cytoskeleton changes*)

**Table 2:** Input final variables, type of input and information related to the labels and metrics.

		Type	Min-Max or Labels
**Exposure condition**	Dose	Numeric	0.001–800 (μgr/mL)
	Duration		1–168 (h)
**Physico-chemical properties**	Nanoparticle	Nominal	FeO, SiO_2_, TiO_2_, Ag, CuO, ZnO, GO, MnO, SWCNT,
	Shape		Spherical, irregular, prism, cubic, nanotube, flat, oval, rod, crystalline, unknown
	Zeta Potential	Numeric	−49–44 (mV), unknown
	Hydro_size		14–2181 (nm), unknown
	Primary size		1–219 (nm)
	Surface Area		17–240 (m^2^/g), unknown
**In vitro character-ristics**	Cell origin	Nominal	Human, rat, mouse
	Cell type		Endothelial, astrocytes, microglial, medulloblastoma, neuroblastoma, mesencephalic, pheochromocytoma, cerebellar granule, Schwann cells
	Cell line		HCMEC, BMEC, primary, ALT, D384, SHSY5Y, N9, BV2, PC12, N2a, CGC, RSC96, N27
	Assay	Nominal	MTT, MTS, XTT, AlamarBlue, LDH, Caspase 3/7, clonogenic, CCK-8, Trypan-blue, PI, BrdU, TUNEL, NRU, Annexin_V/PI
**Output**	Cell viability	Nominal	Toxic, non-toxic

**Table 3:** Internal 10-fold cross validation model performance and external validation-predictivity for the ten random samples of the dataset.

	Internal Validation							
	ACC	PREC	SENS	SPEC	F1	MCC	ROC	
Replication 1	96.7%	96.8%	96.7%	96.8%	0.97	0.94	0.99	**Weighted Avg**
Replication 2	97.6%	97.6%	97.6%	97.6%	0.98	0.95	0.99	
Replication 3	97.9%	97.9%	97.9%	97.9%	0.98	0.96	1.00	
Replication 4	97.5%	97.5%	97.5%	97.5%	0.98	0.95	0.99	
Replication 5	97.6%	97.6%	97.6%	97.6%	0.98	0.95	1.00	
Replication 6	97.4%	97.4%	97.4%	97.4%	0.97	0.95	1.00	
Replication 7	98.1%	98.1%	98.1%	98.1%	0.98	0.96	1.00	
Replication 8	98.0%	98.0%	98.0%	98.0%	0.98	0.96	1.00	
Replication 9	97.9%	97.9%	97.9%	97.9%	0.98	0.96	1.00	
Replication 10	98.2%	98.2%	98.2%	98.2%	0.98	0.96	1.00	
**Average**	**97.7%**	**97.7%**	**97.7%**	**97.7%**	**0.98**	**0.95**	**1.00**	
	**External Validation**							
	**ACC**	**PREC**	**SENS**	**SPEC**	**F1**	**MCC**	**ROC**	
Replication 1	98.5%	98.5%	98.5%	98.4%	0.99	0.97	1.00	**Weighted Avg**
Replication 2	98.4%	98.5%	98.4%	98.5%	0.98	0.97	0.99	
Replication 3	96.2%	96.2%	96.2%	96.1%	0.96	0.92	0.99	
Replication 4	99.2%	99.2%	99.2%	99.3%	0.99	0.98	1.00	
Replication 5	98.5%	98.5%	98.5%	98.4%	0.99	0.97	1.00	
Replication 6	99.2%	99.2%	99.2%	99.0%	0.99	0.98	1.00	
Replication 7	96.9%	97.0%	96.9%	97.0%	0.97	0.94	0.97	
Replication 8	98.4%	98.5%	98.4%	98.4%	0.98	0.97	1.00	
Replication 9	99.1%	99.1%	99.1%	98.7%	0.99	0.98	1.00	
Replication 10	98.3%	98.3%	98.3%	98.3%	0.98	0.97	1.00	
**Average**	**98.3%**	**98.3%**	**98.3%**	**98.2%**	**0.98**	**0.97**	**0.99**	
	**Reliability Validation**							
	**ACC**	**PREC**	**SENS**	**SPEC**	**F1**	**MCC**	**ROC**	
Replication 1	72%	72%	71%	66%	0.72	0.38	0.73	

**Table 4:** Percentage of test set instances falling within the model AD according to different AD methods.

AD Method	% of Test Set Falling within the Model AD
Bounding Box	100
Bounding Box PCA	100
Leverage	100
Distance from centroid	100
Distance kNN—fixed k	100
Distance kNN—variable k	100

**Table 5:** In silico tools available in the literature capturing neurotoxicity. Data source and number of studies regarding data compilation, its size for all tissues, level of biological organization, and final input variables used to predict an endpoint. The algorithm implemented is also shown.

Ref.	Data Availabi-lity	Data Source	Dataset Size and Input Variables	NPs Category	Level of Biological Organisation	Endpoint—Metric	Algorithm Category
[25]	Data spreadsheet provided	Literature: 307	1741 rows. 14 input	Cadmium-containing quantum dots	In vitro	Cell viability and IC50 Numerical	Decision Tree, RF
[24]		Literature: 517	3028 rows cell viability and 837 IC50. 18 input				Bayesian, BN
[26]		Literature: 32	559 rows. 20 input	Metal, Metal oxide	In vivo, in vitro	Aggregated (cytotoxicity, neurological, pulmonary, fibrosis, etc.) (nominal)	
[21]	Not Available	Database (S2NANO): 63	2005 rows. 14 input	Metal (Au, Ag)	In vitro	Cell viability (Binary)	Instance Based, Decision Tree, SVM, RF
[23]		Database (S2NANO): 216	6842 rows. 15 input	Metal, Metal oxide			Decision Tree, RF
[22]	Data spreadsheet provided	Literature: 93	3 datasets: 1052 rows, 1261 rows 540 rows. 17 features	Carbon-based, Metal, Metal Oxide, Polymeric, Dendrimers, Quantum Dots			Decision Tree, DT
[20]	Data spreadsheet provided	Literature: 24	246 rows. 12 input	Metal, Metal oxide, Polymeric		Disrupted processes (i.e., cell cycle and proliferation) (Binary)	Bayesian, BN

**Table 6:** In silico available tools in the literature capturing neurotoxicity (relevant features in bold). The final input variables selected for model implementation are categorized in theoretical descriptors, p-chem properties, exposure attributes and in vitro characteristics.

	Input Variables			
Ref.	Theoretical Descriptors	P-Chem Properties	Exposure Attributes	In Vitro Characteristics
[25]	-	Source, core, shell, diameter, surface ligand, surface charge, surface modification	Exposure dose and time	Cell anatomical type (epithelial, **neuronal**, **neuroblast** etc.), cell origin (cell line, primary), assay type (MTT, MTS etc.), delivery type (passive, active)
[24]		Shell, core, source, diameter, surface ligand, surface charge, surface modification, ligand chemical	Exposure dose and time, delivery type	Cell source species (human, rat etc.), anatomical type (epithelial, **neural**, **neuronal** etc.), identification (**PC12**, **Neuro-2a**, **N9** etc.), tissue organ origin (brain, adrenal gland, hypothalamus etc.), assay type (MTT, LDH etc.)
[26]		Shape, NP type, dissolution, surface area, surface charge, coating, surface reactivity, aggregation, particle size	Administration route	Study type (in vitro or in vivo)
[21]		NPs type, shape, core size, hydrodynamic size (W), surface charge (W), specific surface area, coating	Exposure dose and time	toxic assay method (MTT, MTS, etc.), cell lines (A549, **SHSY5Y**, **BMEC** etc.), cell types (cancer/normal) and cell species (human, murine)
[23]	Quantum Chemical properties	Core size, Surface charge, Hydrodynamic size, Specific surface area, Formation enthalpy, Conduction band energy, Valence band energy, Electronegativity, NP material		Assay (MTT, MTS), cell species (human, mouse, hamster etc.), cell origin (lung, blood, skin, **brain** etc.), cell type (normal/cancer)
[22]	-	NP type, core, surface coating, diameter, surface charge		Cell-type (L929, **HCMEC**, **SHSY5Y** etc.), cell line (primary), Human/animal, animal (mouse, rat etc.), organ/tissue source (**bone marrow**, **brain**, colon etc.), morphology (epithelial, neuronal etc.), test (MTS, MTT etc.), test indicator (tetrazolium salts annexin V etc.), biochemical metric (cell membrane integrity, LHD leakage etc.)
[20]	-	NPs type, core size, shape, coatings, surface area, zeta potential		cell line (HEPG2, VSMC, HACAT etc.), type (cancer, normal), tissue (kidney, **brain**, lung etc.), assay (Affymetrix, bioscience)

**Table 7:** In silico available tools in the literature capturing neurotoxicity. Attribute importance methodology along with the top five and lowest five attributes are shown for relevant outcomes (in bold). In addition, applicability domain and performance metrics and results are shown.

Ref.	Attribute Importance Method	Top 5 Important Attributes	Lowest 5 Important Attributes	Applicability Domain	Performance
[25]	Random Permutation	**IC50:** Diameter>surface ligand>shell>assay type>exposure time. **Cell viability:** Diameter>Concentration>surface ligand>exposure time>surface modification.	**IC50:** cell species<delivery type<cell origin<surface charge<NP source. **Cell viability:** cell anatomical type<cell species<cell origin<delivery type<NP source.	-	Cell viability: R2 = 0.70. IC50: R2 = 0.80
[24]	Sensitivity analysis	**Cell viability:** diameter>concentration>exposure time>surface ligand>assay type. **IC50:** diameter>exposure time>surface ligand>shell>assay type.	**Cell viability:** cell identification<ligand chemical<surface modification<source species<cell anatomical type **IC50:** cell identification<Tissue organ<source species<surface modification<Source	-	(cross) Cell viability: F1 = 0.86 IC50: R2 = 0.87
[26]		Nanoparticle>surface coatings>surface area>Aggregation>Particle size>surface charge>shape>surface reactivity>dissolution		-	(no cross) ACC: 72%
[21]	Weights by chi square statistic method.	Dose>cell line>core size>surface charge>specific surface area.	Shape<species<type of cells<material<time<assay	k-nearest neighbours’ algorithm—weighted Euclidean distance	(only external) Dataset with best results: ACC: 87.0%, SENS: 61.3%, F1 score: 65.2%
[23]	Leave-one-out out-of-bag (OOB) errors	Dose>assay>time>surface area>core size.	Cell type<surface charge<3 quantum chemical properties (Ec<x<Ev)		(cross) Dataset with best results: ACC: 96%, F1: 93%
[22]	Gain ratio algorithm	Not specified, generation of several decision trees		-	(cross) Generation of several decision trees
[20]	Sensitivity analysis	Normalized information gain for each model outcome. In general NP type, exposure dose and in vitro characteristics ranked first among the other variables.		-	Three different models with 9 different outcomes.

## Supplementary Materials

Supplementary materials can be found at https://www.mdpi.com/1422-0067/21/15/5280/s1.
